# 1. Reduced utilization of meropenem post successful implementation of Neonatal Intensive Care Unit empiric sepsis treatment guideline

**DOI:** 10.1017/ash.2025.252

**Published:** 2025-09-24

**Authors:** Laura Sass, Sarah Parsons, Suzanne Quesnel

**Affiliations:** 1 Children’s Hospital of The King’s Daughters

## Abstract

**Background:** Neonatal intensive care units (NICU) are associate with a high level of antibiotic consumption. Appropriate antibiotic use is crucial to minimize the emergence of resistance and unintended consequences to the patient. Our antimicrobial stewardship program (ASP) performed a baseline review of NICU antibiotic prescribing, which revealed excessive meropenem use and inconsistent empiric antibiotic prescribing practices within the unit. Third generation cephalosporins were vastly underutilized due to concerns of increased Candida infections resulting in the unwarranted excessive use of meropenem.1 **Methods:** In 2023, the ASP created an institution specific empiric NICU sepsis guideline to align empiric prescribing practices with current guidelines and reduce the unwarranted use of carbapenems. After education and guideline implementation, a retrospective review, pre (April 16, 2021 to April 16, 2023) and post (April 17, 2023 to April 17, 2024) implementation was conducted. The primary objectives were to evaluate the effect of the guideline implementation on antibiotic days of therapy (DOT) per 1000 patient-days, overall meropenem and third generation cephalosporin utilization, differences in the incidence of Candida infections, and variations in antimicrobial sensitivity. Microbiologic data from sterile site cultures were obtained April 2021 to March 2023 and post-implementation (April 2023 to March 2024) to evaluate cephalosporin and meropenem resistance for each period. **Results:** Meropenem DOT/1000 patient-days declined from 3.9 to 2.0 (51.3%), and an associated rise in third-generation cephalosporin DOT/1000 patient-days from 15.7 to 22.9 (69.7%) occurred post-guideline implementation. There were no observed differences in the incidence of *Candida* infections, cephalosporin resistance in Gram-negative bacilli, or the organisms isolated over the observation period. **Conclusions:** Guideline implementation safely and successfully reduced the use of carbapenems by providing alternative antibiotic regimens encouraging the use of third generation cephalosporins and reduced antibiotic pressure in our NICU. There were no differences in the incidence of Candida infections, organisms, or resistance patterns. Implementation of this guideline resulted in safe decreases in antibiotic use in the NICU.

Cotton CM, McDonald S, Stoll B, et al. The association of third-generation cephalosporin use and invasive candidiasis in extremely low birth-weight infants. Pediatrics. 2006;118(2):717-22.

